# Impact of *Lactobacillus reuteri* colonization on gut microbiota, inflammation, and crying time in infant colic

**DOI:** 10.1038/s41598-017-15404-7

**Published:** 2017-11-08

**Authors:** Monica L. Nation, Eileen M. Dunne, Shayne J. Joseph, Fiona K. Mensah, Valerie Sung, Catherine Satzke, Mimi L. K. Tang

**Affiliations:** 1 Murdoch Children’s Research Institute, Parkville, 3052 Australia; 2 The Royal Children’s Hospital, Parkville, 3052 Australia; 3 The University of Melbourne, Parkville, 3052 Australia

## Abstract

Infant colic is a distressing condition of unknown etiology. An aberrant gastrointestinal microbiota has been associated, and *Lactobacillus reuteri* supplementation has been shown to reduce crying and/or fussing time (‘crying time’) in some infants with colic. The relationship between *L*. *reuteri* gut colonization and crying time has not been examined. We investigated the relationship between *L*. *reuteri* colonization and fecal microbiota (microbial diversity and *Escherichia coli*), intestinal inflammation, and crying time in infants with colic, using a subset of 65 infants from the Baby Biotics trial, which randomized healthy term infants aged <13 weeks with infant colic to receive probiotic *L*. *reuteri* DSM 17938 (1 × 10^8^ colony forming units) or placebo daily for 28 days. We observed an overall reduction in median crying time, regardless of *L*. *reuteri* colonization status (n = 14 colonized). There were no differences in *E*. *coli* colonization rates or densities, microbial diversity or intestinal inflammation by *L*. *reuteri* colonization status. We found that *L*. *reuteri* density positively correlated with crying time, and *E*. *coli* density negatively correlated with microbial diversity. As density of *L*. *reuteri* was associated with increased crying time, *L*. *reuteri* supplementation may not be an appropriate treatment for all infants with colic.

## Introduction

Infant colic is a distressing condition characterized by crying and/or fussing of unknown cause that affects up to 28% of infants under three months of age^1^. The etiology of infant colic is unclear and there is no widely accepted or effective treatment^2,3^. Several studies have found differences in gastrointestinal (GI) microbial diversity between infants with colic and healthy infants, suggesting that an aberrant microbial intestinal profile may cause or contribute to infant colic^4–6^. Infants with colic have higher rates and densities of *Escherichia coli* and other gas-producing coliforms and lower levels of *Lactobacillus* spp. compared with healthy infants^4–7^, with some aerobic bacterial genera not detected in infants with colic^8^. One study found that infants with colic had lower microbial diversity and elevated fecal calprotectin (a marker of gut inflammation) compared with healthy infants^9^. However, another study found no difference in fecal calprotectin levels between healthy infants and infants with colic^10^.

The finding that infants with colic have an altered intestinal microbiota has led to the investigation of probiotic supplementation for the treatment of this condition, with the aim of promoting a healthy intestinal microbiota and reducing intestinal inflammation. Several clinical trials suggest *Lactobacillus reuteri* may reduce crying and/or fussing time (referred to as ‘crying time’) in some infants with colic^11–15^. In contrast, we recently reported results of a double blind, randomized, placebo-controlled trial evaluating *L*. *reuteri* supplementation for the treatment of infant colic in Melbourne, Australia (Baby Biotics trial, Current Controlled Trials ISRCTN95287767 (25/10/2010)) that found no effect on crying time when compared to placebo^16^. However, analysis of fecal samples indicated that less than half of the infants in the probiotic treatment group were colonized with *L*. *reuteri* at day 28 of the trial, and this low colonization rate may have contributed to the negative trial findings. As probiotics are thought to exert their beneficial effects on the host through transiently colonizing the GI tract, we sought to examine the relationship between *L*. *reuteri* colonization and key microbiological, immunological, and clinical characteristics of infant colic. We also investigated the role of *E*. *coli*, which has been implicated in infant colic^4,7,17^. The use of quantitative real-time PCR (qPCR) for detection of *L*. *reuteri* and *E*. *coli* enabled density-based analysis of bacterial loads. This study examined the relationships between *L*. *reuteri* and *E*. *coli* colonization, microbial diversity, intestinal inflammation, and crying time in a subset of infants from the Baby Biotics trial cohort. We hypothesized that infants colonized with *L*. *reuteri* would have lower *E*. *coli* colonization, greater microbial diversity, lower intestinal inflammation (calprotectin levels), and lower crying time, when compared to infants not colonized with *L*. *reuteri*.

## Results

Sixty-five infants (31 probiotic, 34 placebo) were included in this study. Fecal samples were collected on day 28 of treatment and examined for *L*. *reuteri* and *E*. *coli* colonization, microbial diversity, and calprotectin. Some infants were excluded from analysis of diversity, calprotectin, and/or crying time due to no detection of a 16S rRNA gene PCR product or >10% of peak profile consisting of large fragments (*Alu*I, n = 20; *Sau*96I, n = 10), insufficient sample volume (n = 7), or insufficient crying time data (n = 9), respectively.

The two groups in this study, infants colonized by *L*. *reuteri* (n = 14) and infants not colonized by *L*. *reuteri* (n = 51), had similar clinical characteristics (Table 1). The clinical characteristics were also representative of the original Baby Biotics trial cohort, except for higher rates of family history of allergic disease in this study.

**Table 1:** Clinical characteristics of the study population.

Characteristic	Original trial^16^		This study		
	Probiotic (n = 85)	Placebo (n = 82)	Colonized by *L*. *reuteri* (n = 14)	Not colonized by *L*. *reuteri* (n = 51)	P-value^a^
**Gender** Male, n (%)	37 (44)	48 (59)	6 (43)	30 (59)	0.29^b^
**Mode of birth** Caesarean section, n (%)	35 (41)	32 (39)	4 (29)	20 (40)	0.46^b^
**Method of feeding** Exclusively breastfed, n (%)	33 (39)	35 (43)	6 (43)	17 (33)	0.51^b^
**Family history of allergic disease** Yes, n (%)	51 (60)	50 (61)	11 (79)	41 (80)	0.88^b^
**Birth weight (g)** Mean ± SD	3272 ± 406	3426 ± 421	3425 ± 482	3312 ± 398	0.57^c^
**Infant age at enrolment (weeks)** Mean ± SD	7.5 ± 2.9	6.9 ± 2.5	8.2 ± 2.8	7.4 ± 2.7	0.29^c^
**Infant crying time at day 0 (min/day)** ^d^ Mean ± SD	327.6 ± 151.9	329.3 ± 126.4	337.2 ± 186.3	344.1 ± 124.1	0.94^c^

^a^Differences assessed for infants in the current study (colonized versus not colonized by *L*. *reuteri*). ^b^Chi-Squared test; ^c^ Mann-Whitney U test; ^d^Calculated using probiotic (n = 75), placebo (n = 65), colonized by *L*. *reuteri* (n = 13), and not colonized by *L*. *reuteri* (n = 47) due to missing data.

### *L. reuteri* and *E. coli* colonization

**Figure 1:**
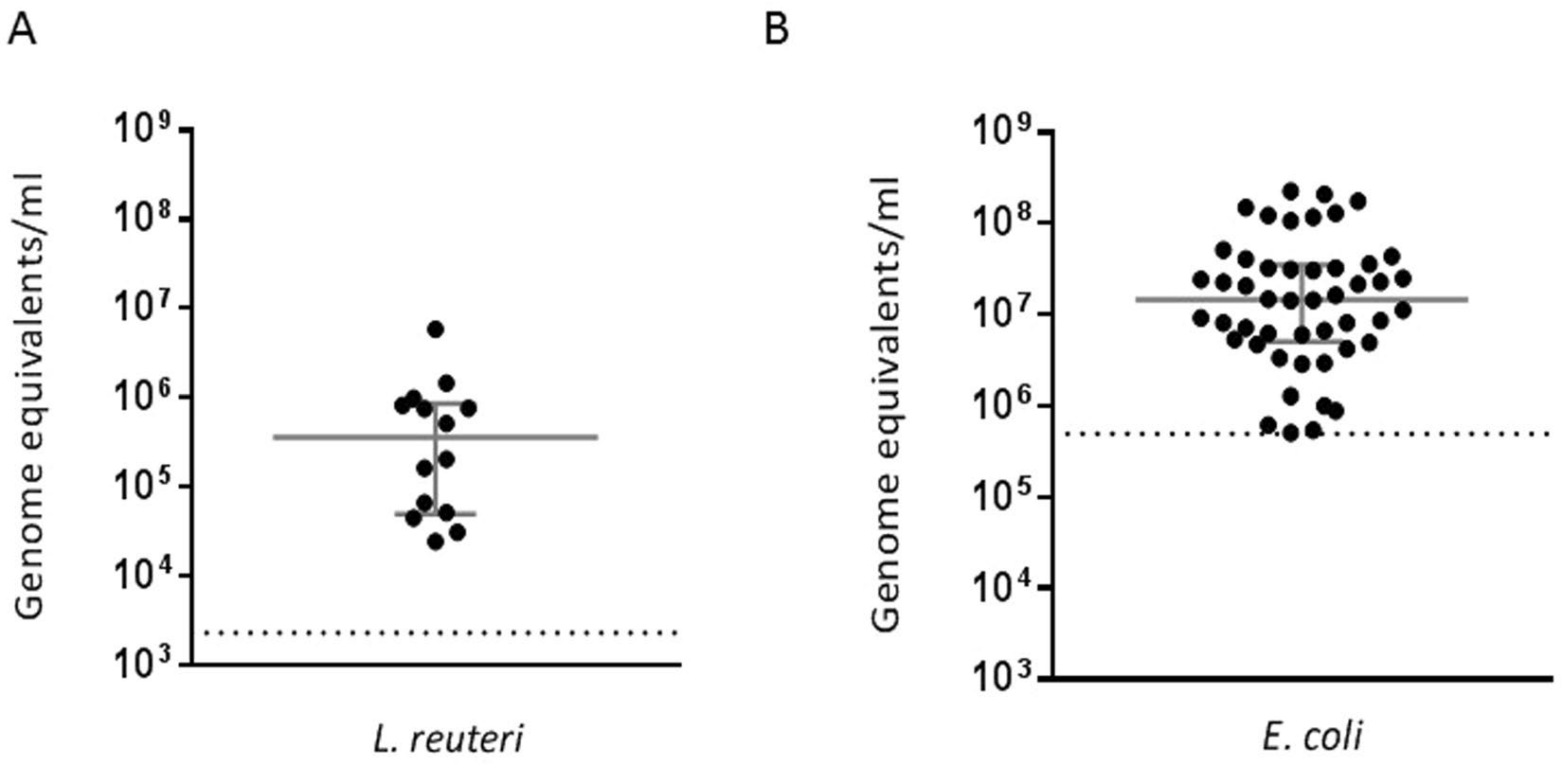
(**A**) *L*. *reuteri* (n = 14) and (**B**) *E*. *coli* (n = 48) colonization levels in fecal samples collected at day 28 from infants with colic. Bars represent median ± interquartile range; dotted lines represent assay limit of detection.

Of the 31 infants that received probiotic, 14 (45%) were colonized with *L*. *reuteri* at day 28, whilst none of the 34 infants in the placebo group became colonized (Chi-squared test, P < 0.0001). Of the infants colonized by *L*. *reuteri*, 93% (13/14) were also colonized by *E*. *coli*, whereas 69% (35/51) of infants not colonized by *L*. *reuteri*, were colonized by *E*. *coli*, however this difference was not statistically significant (Chi-squared test, P = 0.07). Infants colonized with *L*. *reuteri* had a median colonization density of 3.6 × 10^5^ genome equivalents/ml (Interquartile range (IQR): 5.0 × 10^4^, 8.7 × 10^5^ genome equivalents/ml), while infants colonized with *E*. *coli* had a median colonization density of 1.5 × 10^7^ genome equivalents/ml (IQR: 5.0 × 10^6^, 3.5 × 10^7^ genome equivalents/ml) (Fig. 1). There was no difference in *E*. *coli* density in infants colonized or not colonized by *L*. *reuteri* (Table 2). In infants colonized by both species, there was no association between *L*. *reuteri* and *E*. *coli* densities (Spearman’s rank correlation coefficient, r = 0.16; P = 0.60).

**Table 2:** Outcomes by *Lactobacillus reuteri* colonization status, at day 28.

Outcomes	Median (IQR)				P-value^a^
	n	Colonized by *L*. *reuteri*	n	Not colonized by *L*. *reuteri*	
**Fecal** ***E***. ***coli*** **colonization** (genome equivalents/ml)	13	1.4 × 10^7^ (3.6 × 10^6^, 6.9 × 10^7^)	35	1.6 × 10^7^ (5.4 × 10^6^, 3.6 × 10^7^)	0.71
**Fecal microbial diversity score**					
- *Alu*I peaks	10	28.5 (20.5, 35.8)	35	24.0 (21.0, 27.0)	0.14
- *Sau*96I peaks	14	32.0 (28.0, 35.3)	41	31.0 (27.0, 36.0)	0.62
**Fecal calprotectin** (µg/g)	13	135.4 (89.5, 568.5)	45	114.8 (67.1, 237.9)	0.37
**Infant crying time** (min/day)	13	165.0 (100.3, 268.8)	45	180.0 (136.3, 274.1)	0.72

^a^Mann-Whitney U test.

### Microbial diversity

Terminal restriction fragment length polymorphism (T-RFLP) was conducted, with peak profiles generated from FAM-labelled PCR product targeting total 16S rRNA genes using two restriction enzymes (*Alu*I and *Sau*96I) and analyzed separately. Peaks were quantified to provide an estimate of intestinal microbial diversity, with a higher number of peaks representing greater diversity. There was no difference between the number of peaks in infants colonized with *L*. *reuteri* when compared to those not colonized using *Alu*I or *Sau*96I (Table 2). In infants colonized by *L*. *reuteri*, there was no association between density of *L*. *reuteri* colonization and number of peaks in profiles generated by either *Alu*I (Spearman’s rank correlation coefficient, r = −0.52; P = 0.13) or *Sau*96I digestion (Spearman’s rank correlation coefficient, r = 0.26; P = 0.36).

### Intestinal inflammation

Fecal calprotectin levels were measured to investigate inflammation within the GI tract. All 58 infants assessed had detectable levels of calprotectin (median 116.2 µg of calprotectin per g wet feces (µg/g), IQR: 72.3, 255.8). There was no difference in calprotectin levels between infants colonized with *L. reuteri* compared to those not colonized (Table 2). In infants colonized by *L. reuteri*, there was no association between density of *L. reuteri* colonization and calprotectin (Spearman’s rank correlation coefficient, r = 0.16; P = 0.60).

### Crying time

**Figure 2:**
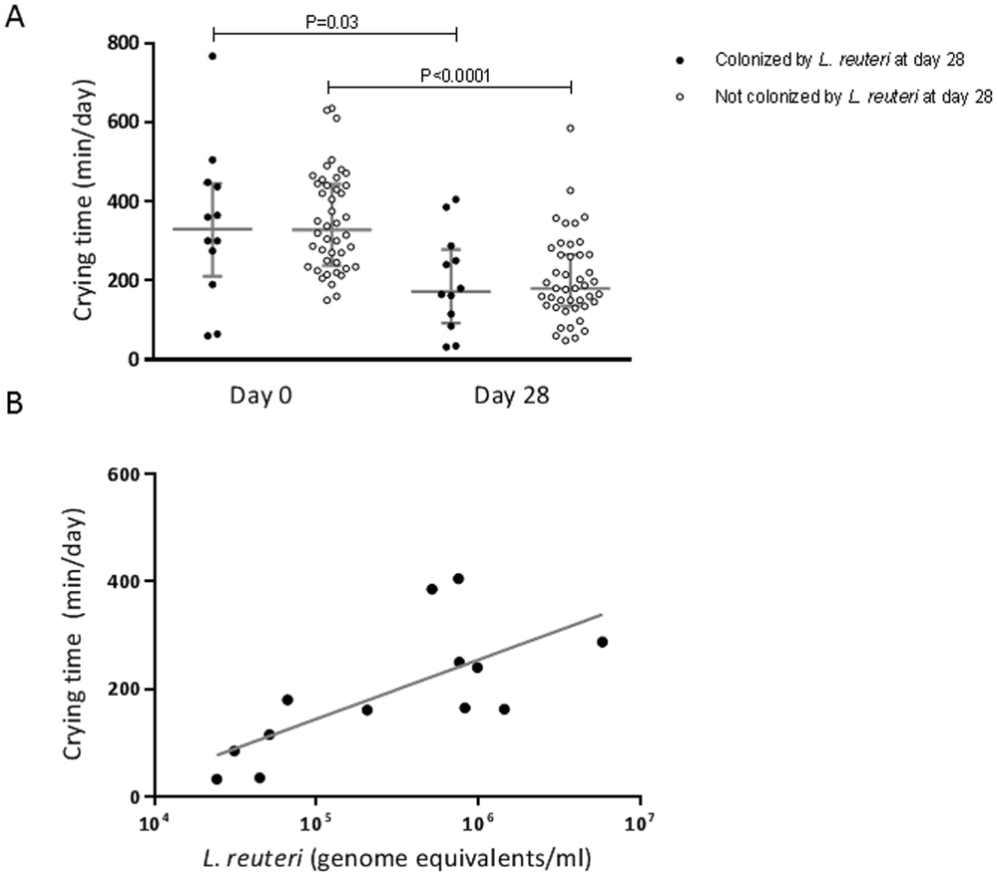
The association between *L*. *reuteri* colonization and infant crying time. (**A**) Crying time in infants colonized (n = 12) and not colonized (n = 44) by *L*. *reuteri*. Infants were categorized as colonized or not colonized by *L*. *reuteri* at both time points by *L*. *reuteri* colonization status at day 28. Bars represent median ± interquartile range. Differences within groups (day 0 to day 28) were assessed using Wilcoxon signed-rank test and differences between groups (colonized and not colonized) using Mann-Whitney U test. (**B**) The relationship between crying time and *L*. *reuteri* colonization density (n = 13); Spearman’s rank correlation coefficient, r = 0.68; P = 0.01.

There was a reduction in infant crying over time, regardless of *L*. *reuteri* colonization status. In infants colonized by *L*. *reuteri*, median crying reduced from 330.0 min/day (IQR: 211.3, 445.3 min/day) on day 0 to 172.5 min/day (IQR: 92.7, 278.1 min/day) on day 28 (Wilcoxon signed-rank test, P = 0.03) (Fig. 2A). Similarly, median crying reduced from 328.4 min/day (IQR: 237.7, 443.8 min/day) on day 0 to 180.0 min/day (IQR: 135.6, 265.5 min/day) on day 28 in infants not colonized by *L*. *reuteri* (Wilcoxon signed-rank test, P < 0.0001). At day 28 there was no difference between median infant crying time between infants colonized and infants not colonized by *L*. *reuteri* (Table 2). For the 13 infants colonized by *L*. *reuteri*, a positive association was found between *L*. *reuteri* colonization density and median infant crying time (Spearman’s rank correlation coefficient, r = 0.68; P = 0.01) (Fig. 2B).

### Secondary analyses

**Figure 3:**
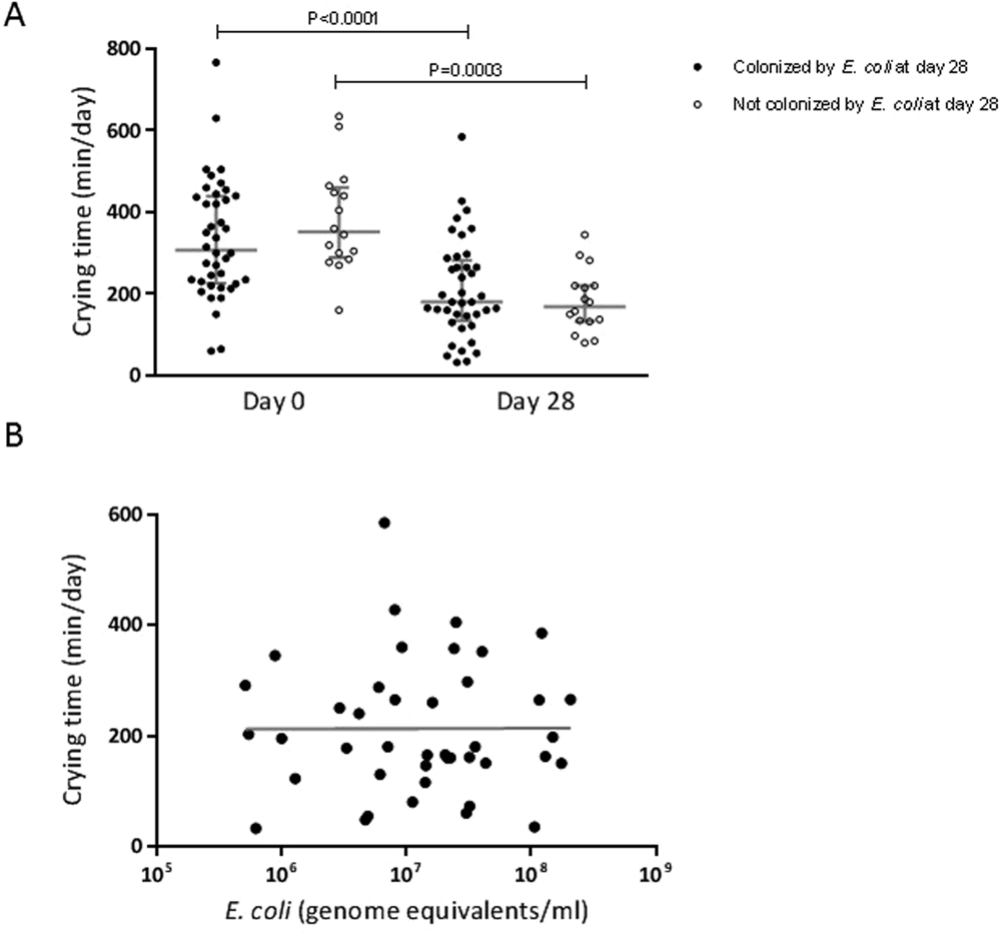
The association between *E*. *coli* colonization and crying time. (**A**) Crying time in infants colonized (n = 40) and not colonized (n = 16) by *E*. *coli*. Infants were categorized as colonized or not colonized by *E*. *coli* at both time points by *E*. *coli* colonization status at day 28. Bars represent median ± interquartile range. Differences within groups (day 0 to day 28) were assessed using Wilcoxon signed-rank test and differences between groups (colonized or not colonized) using Mann-Whitney U test. (**B**) The relationship between crying time and *E*. *coli* colonization density (n = 42); Spearman’s rank correlation coefficient, r = 0.01; P = 0.94.

We additionally assessed the relationships between *E*. *coli* colonization and microbial diversity, intestinal inflammation and crying time (Table 3). We found no differences in microbial diversity in infants colonized or not colonized by *E*. *coli*, and no association between density of *E*. *coli* colonization and number of peaks by *Alu*I digestion (Spearman’s rank correlation coefficient, r = −0.12; P = 0.49). However, in infants colonized by *E*. *coli*, there was a negative association between *E*. *coli* colonization density and number of peaks in profiles generated by *Sau*96I digestion (Spearman’s rank correlation coefficient, r = −0.33; P = 0.03). We found no association between density of *E*. *coli* colonization and calprotectin (Spearman’s rank correlation coefficient, r = −0.06; P = 0.69). Median crying reduced from 307.5 min/day (IQR: 226.3, 439.2 min/day) on day 0 to 180.0 min/day (IQR: 134.0, 282.0 min/day) on day 28 in infants colonized by *E*. *coli* (Wilcoxon signed-rank test, P < 0.0001). Similarly, median crying reduced from 352.5 min/day (IQR: 288.8, 460.8 min/day) on day 0 to 168.8 min/day (IQR: 133.1, 220.3 min/day) on day 28 in infants not colonized by *E*. *coli* (Wilcoxon signed-rank test, P = 0.0003) (Fig. 3A). There was no statistically significant difference between median change in crying time from day 0 to day 28 between infants colonized (81.3 min/day, IQR: 15.5, 263.6) or not colonized (193.8 min/day, IQR: 125.7, 295.6) by *E*. *coli* (Mann-Whitney U test, P = 0.07). For the 42 infants colonized by *E*. *coli*, no relationship was found between *E*. *coli* colonization density and median infant crying time (Spearman’s rank correlation coefficient, r = 0.01; P = 0.94) (Fig. 3B).

**Table 3:** Outcomes by *Escherichia coli* colonization status, at day 28.

Outcomes	Median (IQR)				P-value^a^
	n	Colonized by *E*. *coli*	n	Not colonized by *E*. *coli*	
**Fecal microbial diversity score**					
- *Alu*I peaks	36	25.0 (21.3, 29.0)	9	22.0 (16.5, 26.5)	0.17
- *Sau*96I peaks	44	31.0 (27.0, 36.0)	11	30.0 (28.0, 33.0)	0.87
**Fecal calprotectin** (µg/g)	46	118.0 (70.2, 267.3)	12	94.2 (74.4, 246.5)	0.58
**Infant crying time** (min/day)	42	180.0 (142.0, 288.5)	16	168.8 (133.1, 220.3)	0.44

^a^Mann-Whitney U test.

## Discussion

We previously reported that supplementation with *L*. *reuteri* DSM 17938 did not reduce crying time in breastfed and formula-fed infants with colic^16^. However, the *L*. *reuteri* colonization rates were lower than expected, potentially affecting the Baby Biotics trial results. In this substudy, we examined the relationship between *L*. *reuteri* colonization and microbiological outcomes (*E*. *coli* colonization and microbial diversity), intestinal inflammation, and crying time in a subset of infants from the trial. *L*. *reuteri* colonization rates (45% at ~ 4 months of age) in the current study were lower than those previously reported in other pediatric studies (92% at ~ 2 months^11^, and 65% at ~3 months^18^). The reduction in colonization rates with increasing infant age suggest age at probiotic administration and/or age at sample collection, as well as the use of different microbiological detection methods (culture-dependent versus culture-independent methods in our study), may affect *L*. *reuteri* detection^11,18^.We found median colonization density of *L*. *reuteri* to be 3.6 × 10^5^ genome equivalents/ml in infants who were colonized with *L*. *reuteri*, which is in line with previous research that found mean *L*. *reuteri* colonization densities of 10^4^–10^5^ CFU/g of feces after supplementation^11,19^. Microbial diversity was similar in infants colonized or not colonized with *L*. *reuteri*, consistent with Roos *et al*. who found that treatment with *L*. *reuteri* did not affect the global composition of the bacterial community^20^. We found no difference in calprotectin levels in infants colonized or not colonized by *L*. *reuteri*, and no association between calprotectin levels and crying time at day 28. However, we previously reported that infants who had a reduction in crying time of at least 50% had lower calprotectin levels, suggesting gut inflammation may be implicated in infant colic, although the precise relationship remains unclear^16^. Crying time decreased in infants from day 0 to day 28 regardless of *L*. *reuteri* colonization status, in line with the natural resolution of infant colic that typically occurs when an infant reaches three to four months of age^21^. There were two infants in the *L*. *reuteri* colonized group with lower crying times at day 0 than were observed in the not colonized group (approximately 60 min/day, versus minimum of 150 min/day). This may have decreased the ability to detect a reduction in crying time from day 0 to day 28 in the infants colonized with *L*. *reuteri*, however we still found a reduction in median crying time. We found no difference in crying time at day 28 between infants colonized or not colonized by *L*. *reuteri*. These findings were consistent with the original Baby Biotics trial results, and in contrast to previous reports that *L*. *reuteri* treatment is effective in reducing crying time in infants with colic when crying is assessed by treatment allocation^11–15^. These divergent findings may reflect differences in geographic location of previous studies (Italy, Poland, China and Canada) and rates of exclusive breastfeeding (majority of infants in previous studies versus 35% in our study). Geography and method of feeding are known to impact on gut microbiota^22–25^, therefore our study emphasizes that results from other settings may not be generalizable to our study population, or to Australian infants in general. In infants colonized by *L*. *reuteri*, we observed an unexpected positive relationship between *L*. *reuteri* density and crying time. Although this finding may generate concerns that high density of *L*. *reuteri* could exacerbate symptoms of colic, there does not appear to be a link with gut inflammation as there was no association between *L*. *reuteri* density and calprotectin. It is plausible that some infants may have an underlying gut environment that is associated with higher crying and also facilitates high-density *L*. *reuteri* growth.

In our study, we found no evidence linking *E*. *coli* to gut inflammation as measured by calprotectin. We found a negative association between *E*. *coli* colonization density and microbial diversity, although this was only detected with one of the enzymes used in the diversity assay. *E*. *coli* has been associated with Crohn’s Disease, with decreases in microbial diversity compared with healthy participants^26,27^, suggesting a possible pathophysiological relationship between GI *E*. *coli* colonization and microbial diversity. Savino *et al*. found that infants who responded to *L*. *reuteri* treatment (a reduction of  ≥50% crying time) had a reduction in fecal *E*. *coli*, so our finding of no difference in *E*. *coli* colonization is consistent with the outcome that treatment with *L*. *reuteri* did not reduce crying time in this study population. There was moderate evidence that infants colonized with *E*. *coli* had a smaller reduction in crying from baseline to day 28, however there was no difference between crying time at day 28 when assessed by *E*. *coli* colonization status. It has been previously reported that *L*. *reuteri* administration was associated with a decreased presence of *E*. *coli*, suggesting a potential negative interaction between these two species^11^. However, we did not observe a negative relationship between *L*. *reuteri* and *E*. *coli*, and in fact, 93% of infants colonized with *L*. *reuteri* also had *E*. *coli* detected. This suggests that the findings of Savino *et al*. may have been due to an indirect rather than direct relationship between the two species, and/or could be another example of differences attributable to geography and feeding methods.

To our knowledge, this is the first study to investigate microbiological changes in infant colic by *L*. *reuteri* colonization status. The participants in our study are representative of help-seeking parents and carers from the general population. Because Australian pediatricians do not deliver primary care, families often present to the emergency department for common childhood concerns, especially for conditions such as colic where the burden typically occurs outside business hours. Additionally, this study included both breast and formula-fed infants and did not restrict the mother’s diet to make the probiotic intervention more generalizable. This is in contrast to the majority of infant colic studies that use populations of exclusively (or predominately) breastfed infants^11–15^, and have mothers or infants on a cow’s milk-free diet^11,12,14^. However, this study had several limitations. Only a subset of samples from the original trial were available for the analyses conducted in this pilot project. We performed laboratory analyses at a single time point at the end of treatment when crying time had already reduced, which may have blunted the ability to detect potential differences linked to crying time. The infant microbiome is highly variable, and it is plausible that transient changes due to probiotic colonization may have occurred earlier. Additionally, while we used robust culture-independent molecular methods to identify *L*. *reuteri* and *E*. *coli*, the qPCR primers and probes may detect other closely related species, such as the closely related *Shigella* spp. for the *E*. *coli* assay^28–30^. Nevertheless, only infants in the probiotic group had detectable *L*. *reuteri*, suggesting our assay was specific for the probiotic strain. Lastly, parents were instructed to deliver the supplement using a spoon or dropper; it is not known whether the route of administration impacted *L*. *reuteri* colonization. Advances in molecular methods and sequencing technology have enabled more detailed characterization of the microbiome including identification of more subtle differences in the bacterial communities present. Employing one of these methods may provide further information on differences in the GI microbiota and greater insight into potential impacts of *L*. *reuteri* treatment on the gut microbiome.

We found no reduction in crying time in infants colonized with *L*. *reuteri* and identified an unexpected positive association between *L*. *reuteri* density and crying time. Our results support the finding of the original Baby Biotics trial that *L*. *reuteri* supplementation did not reduce crying time in infants with colic in Melbourne, Australia. We also found a negative association between *E*. *coli* colonization density and microbial diversity, suggesting a possible pathophysiological relationship between *E*. *coli* colonization and microbial diversity. Our findings suggest *L*. *reuteri* supplementation may not be an appropriate treatment for infants with colic in some geographic settings, and that the impact of such an intervention on intestinal microbiota composition remains unclear. As our study included a small subset of infants from the Baby Biotics randomized trial, additional studies with larger sample size are required to further investigate the role of the gut microbiome in infant colic and the utility of probiotic treatment in different populations.

## Methods

### Study participants and design

The 65 infants included in this study were part of a previously described placebo-controlled trial in Melbourne, Australia that assessed *L*. *reuteri* supplementation as a treatment for infant colic^16,31^. In brief, 167 healthy term infants aged <13 weeks with infant colic (modified Wessel’s criteria, crying and/or fussing for ≥3 hours a day, for ≥3 days over seven days) were randomized to receive five drops of probiotic (1 × 10^8^ colony forming units (CFU)/day *L*. *reuteri* DSM 17938; BioGaia AB, Stockholm, Sweden) or placebo daily for 28 days. A random sample of eight returned probiotic bottles were tested to confirm the probiotic suspension was above the required dose; mean *L*. *reuteri* recovery was 1.8 × 10^8^ CFU/5 drops. The trial was approved by the Human Research Ethics Committee of The Royal Children’s Hospital (HREC 30111), and informed consent was obtained for all participants. 127 infants completed the original trial (67 probiotic, 60 placebo). For the current study, fecal samples were analyzed from the first 65 infants enrolled in the original trial who produced adequate fecal sample volume (31 probiotic, 34 placebo; Table 1). All methods were carried out in accordance with relevant guidelines and regulations. All laboratory analyses were performed blinded.

### Infant crying and/or fussing time

Infant crying and/or fussing time (referred to as ‘crying time’) was measured using the Barr diary and analyzed as described elsewhere^32,33^. Infant crying and fussing times were reported by the primary caregiver and collected over a 24 hour period at baseline, and as an average of 48 hours for day 28 of the study (measured over days 28 and 29). The diaries were adjusted for missing information, with diaries that had ≥30% missing data excluded from analyses at the relevant time point. For diaries with <30% missing data, mean values of crying time were generated using the equation: [episodes of daily cry/fuss bouts]*[1440/(1440 – minutes of missing data)].

### Fecal sample collection and storage

Fecal samples were collected on day 28 of the trial. Samples were collected from the infant’s diaper, transferred to a sterile container, and placed immediately into the caregiver’s freezer. Alternatively, the whole diaper was placed into the freezer. Frozen samples were transported on ice in an insulated container from the caregiver’s freezer to the laboratory within a median of 3 days (range 0–20 days). Once received at the laboratory, diaper samples were transferred into a container. All samples were stored at −80 °C until use (maximum storage of 9 months from collection to DNA extraction).

### Extraction of fecal genomic DNA

On the day of DNA extraction, samples were thawed and homogenized before use. Genomic DNA was extracted using the UltraClean® Fecal DNA Isolation Kit (MO BIO Laboratories, Inc., Carlsbad, USA). Briefly, 0.15 g of homogenized fecal sample was added to a bead/ball mixture containing 0.5 g of 0.1 mm Zirconia/Silica beads (BioSpec Products Inc., Bartlesville, USA) and four 3 mm, undrilled, glass balls (Ajax Finechem Pty Ltd., Taren Point, Australia). DNA isolation was completed following the manufacturer’s protocol from the addition of Solution S1 of the MO BIO kit. If pellets were not formed, the sample was spun in a centrifuge for an additional 3 min at 11,300 x *g* before transferring the supernatant. DNA was eluted in a total volume of 100 µl.

### Quantification of *L. reuteri* and *E. coli*

*L*. *reuteri* and *E*. *coli* were quantified by qPCR using previously published primers and probes targeting the *tuf* and *uidA* genes, respectively^28–30^. Each 25 µl reaction consisted of 2 µl of genomic DNA, 1X Brilliant III Ultra-Fast QPCR Master Mix (Agilent Technologies, Santa Clara, USA), 100 µM probe and either 300 µM of *L*. *reuteri* primers or 100 µM of *E*. *coli* primers. Assays were performed using Stratagene MX3005P qPCR instrument with an initial activation of 95 °C for 3 min followed by 40 cycles of 95 °C for 20 s and 60 °C for 20 s. Reactions were performed in duplicate and the mean cycle threshold (Ct) value calculated. Genomic DNA from *L*. *reuteri* DSM 17938 and *E*. *coli* ATCC 25922 were used for standard curves; a mean Ct of <35 and <34, respectively, were considered positive. The assay detection limits were determined by validation against a panel of related bacteria, and no template controls. Samples with a higher Ct than an extraction control were considered negative. Bacterial density data are reported as genome equivalents/ml (assuming one copy of the target gene per genome, one genome per CFU, and a genome size of 2.0 Mb for *L*. *reuteri* and 5.24 Mb for *E*. *coli*).

### Microbial diversity

Microbial diversity was examined using T-RFLP, using a modified version of a method previously described^34,35^. Briefly, the 16S rRNA gene was amplified using universal primers FAM27f and 519r (Sigma-Aldrich, Castle Hill, Australia) and the HotStarTaq® kit (Qiagen). Each 25 µl reaction contained 5 µl genomic DNA, 1.25 U DNA Polymerase, 1 X PCR Buffer, 2.5 mM MgCl_2_, 200 µM dNTPs, 500 nM fluorescently labelled forward primer and 500 nM reverse primer. PCR cycling conditions were denaturation at 95 °C for 5 min, followed by 25 cycles of 94 °C for 15 s, 50 °C for 15 s, 72 °C for 1 min, and an extension of 50 °C for 1 min 30 s and 72 °C for 6 min. Triplicate reactions were pooled. Samples with a PCR product of 480–540 base pairs were used for further processing. PCR products were purified using a Wizard SV Gel and PCR Clean-Up System column (Promega, Annandale, Australia). T-RFLP fragments were generated using separate reactions for enzymes *Alu*I and *Sau*96I (New England Biolabs, Ipswich, USA). In 25 µl reactions, approximately 50–100 ng of purified PCR product was digested with 5 U of enzyme and 1 X NEBuffer 4 (New England Biolabs) in a water bath (37 °C overnight). PCR products were precipitated and analysed by an AB3730 DNA analyser using AB GeneMapper software (Applied Biosystems, Carlsbad, USA) at the Australian Genome Research Facility, Parkville, Australia. Separate peak profiles were generated for each enzyme. Analysis of T-RFLP peak profiles was performed as described previously^34^.

### Calprotectin

100 mg of fecal sample and 4.9 ml of Extraction Buffer were mixed for 3 min in the Fecal Extraction Device (CALPRO AS, Lysaker, Norway) on a Ratek MPS1 plate shaker. Fecal extracts were stored at 4 °C and used within 5 days. Calprotectin levels were measured by enzyme linked immunosorbent assay (CALPROLAB™, CALPRO AS) according to manufacturer’s protocol. The optical density was measured at 405 nm using an ELx808™ Absorbance Microplate Reader (BioTek) and the KCjunior™ (BioTek) package to determine calprotectin levels.

### Statistical analyses

Analyses were completed using GraphPad Prism version 7.01 for Windows (GraphPad Software, San Diego California USA). The measures of *E*. *coli* colonization, microbial diversity, intestinal inflammation, and crying time were stratified by *L*. *reuteri* colonization status and tested for normality using the D’Agostino-Pearson K-squared test. As the majority of data sets were not normally distributed, non-parametric tests were used throughout. Differences between categorical variables were assessed using the Chi-Squared test. Differences within groups (colonized or not colonized by *L*. *reuteri;* colonized or not colonized by *E*. *coli*) were assessed using the Wilcoxon signed-rank test, and between groups using the Mann-Whitney U test. Correlations were assessed using Spearman’s rank correlation coefficient. All tests were two-tailed and P < 0.05 was considered statistically significant.

### Data availability

The datasets generated during and/or analysed during the current study are available from the corresponding author on reasonable request.
